# Technical Properties and Biological Safety of Reprocessing Technique for a Handpiece of Disposable Pulsatile Lavage Device: An Experimental Study

**DOI:** 10.5704/MOJ.2407.008

**Published:** 2024-07

**Authors:** A Pongkunakorn, M Jutawiriyasakun

**Affiliations:** Department of Orthopaedic Surgery, Lampang Hospital, Lampang, Thailand

**Keywords:** recycling, therapeutic irrigation, pulsatile flow, arthroplasty

## Abstract

**Introduction:**

Bony surface cleaning using a pulse lavage device (PLD) is essential for modern cementation of hip and knee arthroplasties. This costly single-use device is a medical waste and unaffordable for some patients. Reprocessing is a worldwide standard strategy to solve this problem. To determine the technical properties and biological safety of a reprocessed PLD handpiece and compare its performance under different power supplies.

**Materials and Methods:**

Eight brand-new disposable PLDs were tested for baseline technical properties (flow rate, pulse frequency, and peak pressure). Thereafter, they were reprocessed and retested for 10 rounds using two different power supplies. An adenosine triphosphate (ATP) swab test was performed on the PLD accessory parts after cleansing and disinfection. Passed-through isotonic sodium chloride solution ejected from the reprocessed PLD underwent aerobic bacterial culture. The unit costs of production were analysed.

**Results:**

The mean flow rate of the disposable PLD (1.5±0.1 L/min) was less than that of reprocessed PLD using DC15V battery (2.5±0.3 L/min, p<0.001) and AC/DC15V3A adapter (6.1±0.4 L/min, p<0.001). The mean pulse frequency and peak pressure of the disposable PLD and reprocessed PLD using DC15V battery were not different (18.5±0.8 vs 18.8±2.5 Hz, p=0.155 and 0.37±0.04 vs 0.38±0.03 N/mm2, p=0.640, respectively), but were lower than those using AC/DC15V3A adapter (47.0±2.7 Hz, 0.45±0.03 N/mm2, p<0.001). All ATP swab tests, and aerobic fluid cultures yielded negative results. The total cost of reprocessing was 10% of disposable PLD.

**Conclusion:**

A disposable PLD handpiece can be reprocessed without deteriorating its technical properties and used with either retrieved DC15V battery or AC/DC15V3A adapter for the power supply. As the biological safety of reprocessed and disposable PLDs was comparable, it may be clinically utilised with 90% cost reduction.

## Introduction

Modern cementing techniques are crucial for successful outcomes of cemented arthroplasty. Cleaning the bony surface using a pulse lavage device (PLD) is an essential step in modern cementation to remove bone debris, marrow, fat residue, and blood^1^. Pulse lavage or therapeutic irrigation provides better cement interlocking with the cancellous bone^2^ in total knee^3-5^, partial knee^6^, and hip replacements^1,7^ and a significantly lower risk of revision^8^. It also reduces the risk of fat embolism during cement pressurisation^9,10^ and the post-operative infection rate in hip hemiarthroplasty^11^ and may be cost-effective for the prophylaxis of prosthetic joint infection^12^. PLDs are available either fully-disposable or semi-disposable products. The semi-disposable PLD consists of a reusable pneumatic handpiece and a sterile single-use nozzle kit. The pneumatic handpiece is highly expensive and requires a pneumatic connector, an air hose, compressed nitrogen, or an air source with a pressure regulator. Fully-disposable devices are more popular and typically operate using battery packs and spray nozzles. The cost of fully-disposable PLD is 80 USD, accounting for 7% of the total knee prostheses and 10% of the bipolar hip prostheses. However, this device is expensive for routine use in many hospitals with resource constraints. This single-use device is unaffordable for some patients and also considered as a medical waste after using.

In general, two nozzles are available in each disposable PLD package: a fan spray nozzle for knee surgery and a long nozzle for hip surgery. It implies that one nozzle must be left unused and discarded after the completion of each operation. This unused nozzle can be re-sterilised and safely utilised. Moreover, a new nozzle can be purchased as a separate item from the manufacturer. If the PLD handpiece could be reprocessed and reused with a new nozzle, it would be beneficial for many hospitals in terms of economic and environmental advantages.

Reprocessing is a special recycling process in which a material is processed such that it can be reused. The reprocessing of single-use devices (SUDs) is a common practice in many hospitals worldwide^13^, as it is safe, if performed properly, and good for the environment and hospital budgets. This can be performed either in-house or using a licensed reprocessor. In developed nations, expensive and high-tech SUDs are reprocessed only if there is sufficient evidence of safety and effectiveness, and the reprocessing practice is regulated and carefully conducted^14^. Health care providers can reuse SUDs if the facility establishes quality reprocessing^13^.

After the device is used, reprocessing techniques include disassembly, decontamination, cleaning, inspection, testing, packing, relabelling, and sterilisation. We developed a reprocessing technique for a handpiece of a disposable PLD that is practical and can be consistently performed in any general hospital.

This study aimed to evaluate this reprocessing technique in terms of (1) PLD technical properties when receiving different power supplies, (2) biological safety of cleansing procedures and passed-through fluids, and (3) unit costs of production.

## Materials and Methods

This experimental study tested the technical properties of eight brand-new disposable PLDs as baseline data for the control group. These eight PLD handpieces were reprocessed and retested using two types of power supply, including the direct current 15-volt (DC15V) battery retrieving from the disposable PLD, and DC15V 3 amperes from 220V alternating current (AC/DC15V3A) adapter. Thereafter, the samples were repeatedly reprocessed and retested for 10 cycles. In each cycle of reprocessing, biological safety was tested to determine bacterial contamination before and after sterilisation.

The full set of the Cleanest PLD [Guangzhou Clean Medical Products, China] comprised a handpiece (including a DC motor, compression pump, trigger, nozzle lock and two plastic enclosures), nozzle, irrigation tube, waste tube, trocar, battery case, and power wire. It was powered by 10 regular AA batteries (1.5V) that offered low-speed fluid delivery for gentle lavage and a high-speed setting, offering a more powerful cleaning action. The plastic enclosure was held in place using seven small plastic tabs that were snapped together.

**Fig. 1:**
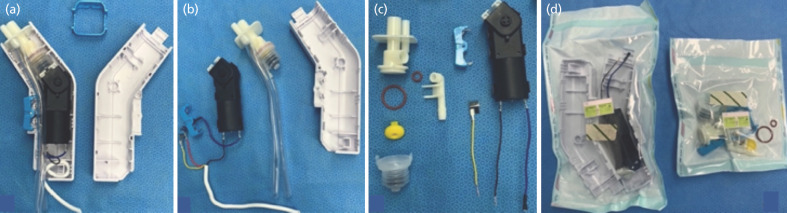
(a) The internal components of the handpiece of pulse lavage device after the plastic enclosures were split, (b) and removed from the housing side of the enclosure. (c) The compression pump was separated into six parts. (d) The motor was disconnected from the trigger. Electric wires were cut and stripped. After the cleansing and disinfection processes, all accessory parts were sterilised.

The reprocessing technique included four steps. Step 1: Disassembly. The power wire, irrigation tube, and waste tube were cut from the handpiece. The plastic enclosures were split by inserting a small flat screwdriver into the middle groove and carefully pried them off (Fig. 1a). The nozzle lock, compression pump, DC motor, and trigger were removed from the housing side of the enclosure (Fig. 1b). The compression pump was separated into six parts (reciprocating bellows, silicone valve, inlet port, pump unit and two silicone o-rings) after the removal of the two water tubes. One electric wire (blue) of the motor was disconnected from the trigger. The other wire (black) of the motor and another yellow wire were cut 5cm from their ends, and the insulation was stripped from the tips (Fig. 1c).

Step 2: Cleansing, disinfection and first sterilisation. The outer surface of the motor and its two electric wires were polished using a soaked 70% ethyl alcohol-cotton ball. Other accessories were sent for cleansing and disinfection. The pre-cleaning process was achieved by foaming a dual-enzymatic spray [EmPower Foam, Metrex, USA] to break down protein-rich dirt or fluid from the instruments. Thereafter, they were soaked in a high-level disinfectant [MetriCide OPA Plus, Metrex, USA], brushed with a non-metallic scrub brush, and flushed using a syringe. All parts were rinsed with pipe water and soaked again in an ultrasonic cleanser [Medisafe Sonic Irrigator, STERIS, USA] for 30 minutes before air-drying with an air blow gun. They were kept in an incubator at 60°C for 30 minutes and sterilised with ethylene oxide (EO) gas (Fig. 1d).

**Fig. 2:**
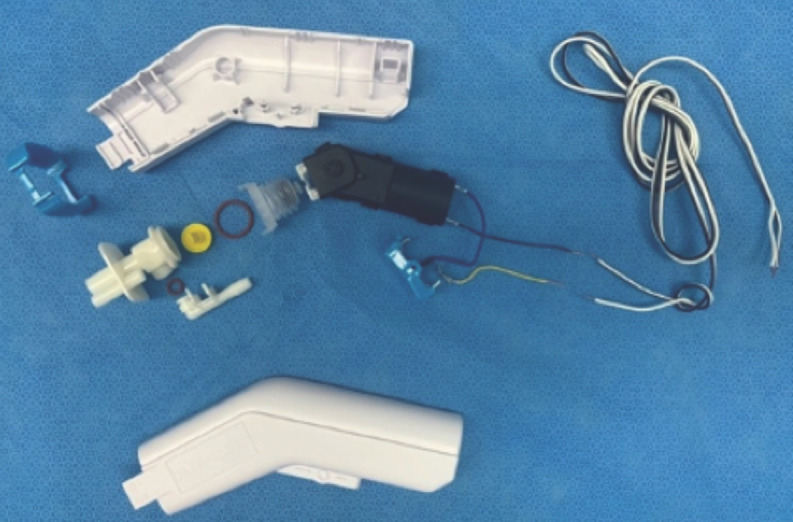
Exploded view in the reassembly step of the pulse lavage device components.

**Fig. 3:**
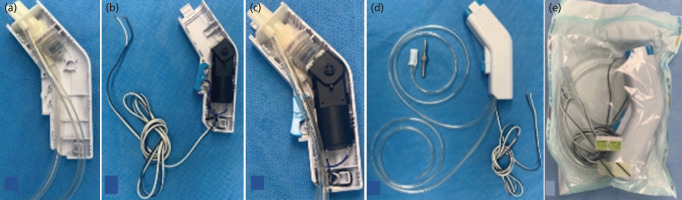
(a) All parts of the water system were packed into the enclosure. (b) The electric part was reassembled to form an open circuit that can be closed by pressing the trigger. (c) After attaching the motor to the bellows, all parts of the electric and water systems were packed together into the enclosure. (d) Two enclosures were snapped together. (e) The reassembled pulse lavage device and a metal trocar were sterilised.

Step 3: Reassembly. This step was performed in the operating room on a table covered with sterile drape. The compression pump was reassembled using a sterile technique, starting by placing the two-silicone o-rings in the inlet port and pump unit. A yellow silicone valve was inserted into the pump unit and closed using the bellows. The inlet port was connected to the pump unit (Fig. 2). Two polyethylene tubes of disposable urine drainage bags (6mm internal diameter, 100cm length) were snugly fitted to the inlet and outlet ports to function as irrigation and waste tubes, respectively. All the parts of the water system were packed in an enclosure (Fig. 3a). The electric part was reassembled by connecting the motor to the trigger and a 200-cm paired-electric cord. This formed an open circuit that could be closed by inwardly pressing the trigger (Fig. 3b). The twinned wires were insulated using a heat-shrink tube before being heating using a hot-air dryer. The driving mechanism of the motor was attached to the bellows (Fig. 3c). The paired-electric cords were looped to form a knot around the wire socket to avoid inadvertent pulling. The covered part of the enclosure was snapped together with the housing (Fig. 3d).

**Fig. 4:**
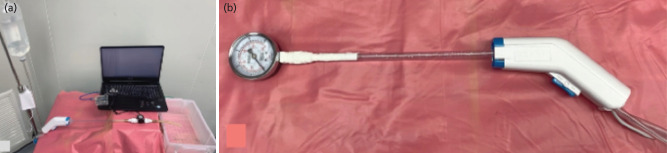
(a) The flow rate and pulse frequency were measured through the water flow meter using a microcontroller board and program. (b) Analog pressure gauge was used for measuring the peak pressure of fluid ejection.

Step 4: Second sterilisation. The reassembled PLD and metal trocar were sterilised using an EO sterilisation chamber and used in the experiment (Fig. 3e). This experiment consisted of the tests for technical properties and biological safety. Tests for technical properties included; (1) test for volumetric flow rate, (2) pulse frequency, and (3) peak pressure of fluid ejection. A disposable PLD irrigation tube was connected to a bag of sterilised isotonic sodium chloride solution using a trocar. For the reprocessed PLD, the irrigation tube was connected to another polyethylene tube in a disposable urine drainage bag (100cm long) to extend its length before receiving a trocar at the tail end. The long nozzle was connected to a handpiece, and its tip was connected to a water flow meter [SEA YF-S201, Kuongshun Electronic, China] using a connecting tube and waterproof sealed with a thread-seal tape. The PLD was connected to the power supply, and the trigger was depressed. The pulsatile flow rate and pulse frequency of the fluid ejected through the water flow meter were computerised using a microcontroller board [Arduino Uno Rev3, Arduino, Italy] and the Arduino IDE program (version 1.8.10) (Fig. 4a). The results were randomly selected for 15 times of 5-second period and the mean value was recorded.

For testing the peak pressure of fluid ejection, the long nozzle was detached from its base, and the internal tube was removed. The remaining outer tube was directly connected to the PLD handpiece, and its tip was connected to an analogue pressure gauge (FIDA PG-71, China) using a metal connector and waterproof-sealed with thread-seal tape (Fig. 4b). The trigger was depressed and the pressure, represented by the rapid movement of the meter needle, was recorded by capturing a digital video for 10 seconds using the video function of a smartphone. Peak pressure was examined using video analysis in slow-motion mode, and the maximum value was recorded as the peak pressure. This process was performed for 10 times, and the average value was used for data analysis.

Tests for biological safety consisted of test to assess efficiency of cleansing procedures and test to identify pathogenic bacteria in the passed-through fluid. For testing an efficiency of cleansing procedures, an adenosine triphosphate (ATP) swab test [3MTM Clean-TraceTM ATP Surface Test, 3M, USA] was performed on the surfaces of the PLD accessories after cleansing, disinfection, and air-drying. This was primarily focused on the inner surface of the bellows and accessory parts with a large lumen (diaphragm, inlet port and outlet port of the pump unit). ATP is an excellent marker of organic and biological contamination. The ATP test can be used to assess equipment sanitation processes and the efficiency of cleansing procedures. Ten tests were performed for 10 rounds of cleansing.

Test to identify pathogenic bacteria in the passed-through fluid aimed to assess the reprocessed PLD after EO sterilisation for bacterial contamination and reprocessing adequacy. Five mL of the passed-through isotonic sodium chloride solution ejected from the pump unit of the reprocessed PLD was stored in a sterile bottle before starting the physical efficacy test. It was sent for aerobic bacterial culture to identify the pathogenic aerobic organisms. The passed-through fluid from the disposable PLD was also used as the control group. A total of 88 specimens were collected and the results were recorded.

The primary study goal was to compare the flow rate of the reprocessed PLD with the disposable PLD in high-speed mode under two different power supplies; (1) DC15V battery case retrieved from the disposable PLD (containing ten 1.5V AA alkaline batteries); and (2) AC/DC15V3A adapter. The secondary study goals were to compare the pulse frequency and peak pressure of the reprocessed PLD with the disposable PLD under two power supplies, and to determine the positive rate of ATP tests and aerobic bacterial cultures of the passed-through isotonic sodium chloride solution. The unit cost of each reprocessing process was also analysed. A paired t-test was used to compare the flow rate, pulse frequency and peak pressure between the disposable and reprocessed PLDs, and among the two power supplies. The Shapiro-Wilk test was used to confirm the normal distribution of the data. The F-test was used to evaluate whether two normal populations have the same variance. Statistical analyses were performed using STATA version 12.1 [StataCorp LLC, College Station, USA]. The level of significance was set at p<0.05.

The sample size was calculated from our pilot study, which found that the mean flow rate of disposable PLD was 1.54 L/min (standard deviation, 0.11). We considered a 10% difference in this flow rate (0.154 L/min) as a clinically relevant effect size for the reprocessed PLD. Using the two dependent means formula with a type I error level of 0.01 and 90% statistical power, the sample size was eight PLD handpieces. The study protocol was approved by our institutional review board.

## Results

The mean flow rate of the disposable PLD was significantly less than that of the reprocessed PLD using a DC15V battery (1.5±0.1 vs 2.5±0.3 L/min, p<0.001), and that of the reprocessed PLD using an AC/DC15V3A adapter (6.1±0.4 L/min, p<0.001). The mean pulse frequencies of the disposable PLD and the reprocessed PLD using a DC15V battery were not different (18.5±0.8 vs 18.8±2.5 Hz, p=0.155), but they were significantly lower than that of the reprocessed PLD using an AC/DC15V3A adapter (47.0±2.7 Hz, p<0.001). Likewise, the mean peak pressures of the disposable PLD and the reprocessed PLD using a DC15V battery were similar (0.37±.0.04 vs 0.38±0.03 N/mm^2^, p=0.640), but they were lower than that of the reprocessed PLD using the AC/DC15V3A adapter (0.45±0.03 N/mm^2^, p<0.001) (Table I).

**Table I:** Technical properties of the PLD in high-speed mode, comparing between the disposable PLD and the reprocessed PLD under two different power supplies.

Technical properties	Disposable PLD (mean±SD)	Reprocessed PLD		p-value
		DC15V battery (mean±SD)	AC/DC15V3A adaptor (mean±SD)	
Flow rate (L/min)	1.5±0.1	2.5±0.3		<0.001
	1.5±0.1		6.1±0.4	<0.001
		2.5±0.3	6.1±0.4	<0.001
Pulse frequency (Hz)	18.5±0.8	18.8±2.5		0.155
	18.5±0.8		47.0±2.7	<0.001
		18.8±2.5	47.0±2.7	<0.001
Peak pressure (N/mm2)	0.37±.0.04	0.38±0.03		0.640
	0.37±.0.04		0.45±0.03	<0.001
		0.38±0.03	0.45±0.03	<0.001

All ATP tests, which assessed 10 rounds of cleansing and sanitation, were negative. The results of the aerobic bacterial culture of the passed-through fluid were negative for all 88 specimens. The total cost of the reprocessed PLD was 8.0 USD. Among these, two-thirds (5.3 USD) were direct costs (three urine bags, a 2-m electric cord, a 10-cm heat-shrink tube, EO sterilisation cost and depreciation cost of the AC/DC adapter), and one-third (2.7 USD) were indirect costs (cleansing and sanitisation services and wages for disassembly and assembly).

With a disposable PLD cost of 80 USD, the total cost of reprocessing each PLD accounted for 10% of the disposable PLD.

## Discussion

The main findings of this study were as follows: (1) reprocessed and disposable PLDs provided similar technical properties using a DC15V battery as power supply, except for a higher flow rate among the reprocessed PLD; (2) the technical properties in three aspects of reprocessed PLD was higher when the power supply was AC/DC15V3A adapter; and (3) sterility and biological safety of reprocessed and disposable PLDs were comparable.

Disposable PLDs available on the market have a variety of flow rates in the high-speed mode, including 0.65 L/min [InterPulse, Stryker, USA], 1.1 L/min [Pulsavac Plus, Zimmer-Biomet, USA], and 1.3 L/min [PalaJet, Heraeus Medical, Germany]^15,16^. In this study, the Cleanest PLD's flow rate was 1.5 L/min, which was higher than that of the brands mentioned. There is currently no standard or recommended flow rate in the literature for the PLD. Although the same DC15V battery was used in 10 rounds, the mean flow rate of the reprocessed PLD was 1.7 times of the disposable PLD. This can be explained using Poiseuille’s law: the flow rate through a tube is inversely proportional to the tube length, and directly proportional to the fourth power of the tube radius^17^. The length of the irrigation tube from the fluid bottle to the PLD handpiece was 200cm in the reprocessed PLD and 300cm for the disposable PLD. The diameter of the irrigation tubes of the reprocessed and disposable PLD was 6mm similarly. The shorter length of the irrigation tube in the reprocessed PLD resulted in the higher flow rate than the disposable PLD in this study.

The mean pulse frequency of 19Hz generated by the disposable PLD did not differ from that generated by the reprocessed PLD using the same DC15V battery. A previous study on disposable PLD using DC batteries reported a mean pulse frequency of 19Hz by the InterPulse PLD and 17–24Hz by the Pulsavac Plus PLD^1,15^. In general, a pulse frequency required by PLD to optimise expulsion of foreign particles is at least 13Hz^16^.

Disposable PLDs available in the market usually use a DC battery power supply in the voltage range of 12V–15V and discard it after a single use. DC battery power depletion is a major concern because DC produces a steady current that easily and gradually loses power over time. The advantages of reusable AC/DC adaptors over batteries are consistency, non-diminishing power output, and eliminating the expense of repacking AA batteries. The reprocessed PLD using the AC/DC15V3A adapter provided flow rates and pulse frequencies as 2.5 times those of the disposable or reprocessed PLD using the DC15V battery because of the higher wattage power required to regulate the motor speed (45 watts by the AC/DC15V3A adapter, and 37.5 watts by the DC15V battery).

The mean peak pressures of the disposable and reprocessed PLD using the same DC15V battery were comparable at 0.37–0.38 N/mm^2^. Utilising AC/DC15V3A adapter as power supply, the reprocessed PLD could significantly increase the peak pressure to 0.44 N/mm^2^. Breusch *et al* demonstrated that the PLD pressure of 0.41 N/mm^2^ (60 psi) up to 16.7Hz could allow cement penetration of 57% of the cross-sectional area of the proximal femur, which is more effective than syringe lavage^1^. Moreover, Knappe *et al*^15^ found that the average impact pressures of 0.38 N/mm^2^ delivered by the Pulsavac Plus PLD and 0.53 N/mm^2^ by the InterPulse PLD could achieve a cleaning depth of 3.7mm and 3.0mm respectively in carbon foam specimens using as substitutes for human cancellous bone. The upper-bound pressure of 0.48 N/mm^2^ appeared to be the best for insuring optimum cancellous bone preparation^16^. Generally, a PLD with a cleaning depth of at least 3mm is recommended for cleaning the cancellous bone in cemented arthroplasty^15,18^. Therefore, the reprocessed PLD using any power supply in this study should reach a cleaning depth of 3mm for the cancellous bone.

All ATP tests assessing the cleansing and sanitation processes were negative. These findings validate the cleanliness of the disassembled parts of the PLD handpiece prior to sterilisation. Similarly, we could not identify any pathogenic aerobic bacteria in the passed-through fluid from the handpieces of eight disposable PLDs and 80 reprocessed PLDs. The current gold standard for evaluating the effectiveness of reprocessing is obtaining bacterial cultures from devices after reprocessing, but before patient use^19^. This can be explained by the effective cleaning and disinfection processes, as well as the double EO sterilisation obtained before and after the reassembly step. MetriCide OPA Plus, used in the disinfection process, is a high-level disinfectant for reprocessing endoscopes and other heat-sensitive, semi-critical medical devices. According to the Hazardous Material Identification System Rating, it is categorised as a health hazard level 1 (irritation or minor reversible injury possible). The chronic hazards are not currently known. Thus, the PLD's accessories were thoroughly cleaned with pipe water following the use of this disinfectant, and then they were resoaked in an ultrasonic cleaner to eradicate any residual of it.

The total cost of a reprocessed PLD was 10% that of a disposable PLD. Among these, two-thirds were direct costs, mainly from the EO sterilisation process and single-use materials. One-third was indirect cost, mainly skilled labour cost during the disassembly and reassembly processes for 40 minutes per piece. The calculated labour cost was based on a daily wage of 24 USD in an upper-middle-income country.

This study had some limitations. First, the experiment was limited to only one design of commercial disposable PLD. Two halves of the plastic enclosures must be capable of manual separation and reassembly using manual compression without deterioration of the locking mechanism. Second, this study was implemented in vitro, and there was no contamination with bone marrow, blood, or bone debris in the operative field. Third, we did not use a digital pressure gauge to measure peak pressure because it was not waterproof. Finally, although several studies showed that PLD gives better cement penetration compared to manual irrigation and can lower the risk for hip revision, there seems to be a lack of evidence that PLD yields a better outcome or implant survival in knee arthroplasty. However, this was the first experimental study to establish a qualified technique for reprocessing relatively expensive and high-tech SUDs in orthopaedics. Our evidence of safety and effectiveness affirms that health care providers can reprocess PLD if this quality process has been achieved.

## Conclusion

The reprocessing technique for the handpiece of disposable PLD demonstrated highly technical properties comparable to that of a new device when the same DC15V battery was the power supply and was more effective when the power supply was an AC/DC15V3A adapter. The sterility and biological safety of reprocessed and disposable PLDs were comparable. This technique is practical and can be performed in most general hospitals. With a 90% reduction in cost, it may be useful in resource-constrained hospitals, where disposable PLDs are too costly to be routinely used in practice.

## References

[1] Breusch SJ, Norman TL, Schneider U, Reitzel T, Blaha JD, Lukoschek M (2000). Lavage technique in total hip arthroplasty: jet lavage produces better cement penetration than syringe lavage in the proximal femur. J Arthroplasty..

[2] Satalich JR, Lombardo DJ, Newman S, Golladay GJ, Patel NK (2022). Cementation in total hip arthroplasty: history, principles, and technique. EFORT Open Rev..

[3] Helwig P, Konstantinidis L, Hirschmüller A, Miltenberger V, Kuminack K, Südkamp NP (2013). Tibial cleaning method for cemented total knee arthroplasty: An experimental study. Indian J Orthop..

[4] Refsum AM, Nguyen UV, Gjertsen JE, Espehaug B, Fenstad AM, Lein RK (2019). Cementing technique for primary knee arthroplasty: a scoping review. Acta Orthop..

[5] Schlegel UJ, Bishop NE, Morlock MM, Nagel K (2015). Comparison of different cement application techniques for tibial component fixation in TKA. Int Orthop..

[6] Seeger JB, Jaeger S, Bitsch RG, Mohr G, Röhner E, Clarius M (2013). The effect of bone lavage on femoral cement penetration and interface temperature during Oxford unicompartmental knee arthroplasty with cement. J Bone Joint Surg Am..

[7] Sharma D, Spacey K, Sharma V, Vince A (2021). Cessation of Pulsed Lavage During the SARS-CoV-2 Pandemic: The Effect on Hip Hemiarthroplasty Cement Mantles. Cureus..

[8] Malchau H, Herberts P, Söderman P, Odén A Prognosis of total hip replacement. Update and validation of results from the Swedish National Hip Arthroplasty Registry 1979-1998. Scientific exhibition presented at: the 67th Annual Meeting of the American Academy of Orthopaedic Surgeons; 2000 March 15-9; Orlando, USA.

[9] Bökeler U, Bühler A, Eschbach D, Ilies C, Liener U, Knauf T (2022). The Influence of a Modified 3rd Generation Cementation Technique and Vaccum Mixing of Bone Cement on the Bone Cement Implantation Syndrome (BCIS) in Geriatric Patients with Cemented Hemiarthroplasty for Femoral Neck Fractures. Medicina (Kaunas)..

[10] Breusch SJ, Reitzel T, Schneider U, Volkmann M, Ewerbeck V, Lukoschek M (2000). Zementierte Hüftendoprothetik--Verminderung des Fettembolierisikos mittels gepulster Druckspülung [Cemented hip prosthesis implantation--decreasing the rate of fat embolism with pulsed pressure lavage]. Orthopade..

[11] Hargrove R, Ridgeway S, Russell R, Norris M, Packham I, Levy B (2006). Does pulse lavage reduce hip hemiarthroplasty infection rates?. J Hosp Infect..

[12] Luck T, Zaki P, Michels R, Slotkin EM (2022). The Cost-Effectiveness of Normal-Saline Pulsed Lavage for Infection Prophylaxis in Total Joint Arthroplasty. Arthroplast Today..

[13] Wang D, Wu J (2019). Reprocessing and reuse of single-use medical devices in China: a pilot survey. BMC Public Health..

[14] Collier R (2011). Reprocessing single-use devices: an international perspective. CMAJ..

[15] Knappe K, Bitsch RG, Schonhoff M, Walker T, Renkawitz T, Jaeger S (2021). Pulsatile Lavage Systems with High Impact Pressure and High Flow Produce Cleaner Cancellous Bone Prior to Cementation in Cemented Arthroplasty. J Clin Med..

[16] Morgan J, Holder G, Desoutter G (2003). The measurement and comparison of jet characteristics of surgical pulse lavage devices. J Arthroplasty..

[17] Pontiga F, Gaytán SP (2005). An experimental approach to the fundamental principles of hemodynamics. Adv Physiol Educ..

[18] Cawley DT, Kelly N, McGarry JP, Shannon FJ (2013). Cementing techniques for the tibial component in primary total knee replacement. Bone Joint J..

[19] Olafsdottir LB, Wright SB, Smithey A, Heroux R, Hirsch EB, Chen A (2017). Adenosine Triphosphate Quantification Correlates Poorly with Microbial Contamination of Duodenoscopes. Infect Control Hosp Epidemiol..

